# A survey of piroplasms in white-tailed deer (*Odocoileus virginianus*) in the southeastern United States to determine their possible role as *Theileria orientalis* hosts

**DOI:** 10.1016/j.ijppaw.2022.05.005

**Published:** 2022-05-19

**Authors:** Alec T. Thompson, Kayla B. Garrett, Megan Kirchgessner, Mark G. Ruder, Michael J. Yabsley

**Affiliations:** a Southeastern Cooperative Wildlife Disease Study, Department of Population Health, College of Veterinary Medicine, University of Georgia, Athens, GA, 30602, USA; b Center for the Ecology of Infectious Diseases, Odum School of Ecology, University of Georgia, 30602, USA; c Warnell School of Forestry and Natural Resources, University of Georgia, Athens, GA, 30602, USA; d Virginia Department of Wildlife Resources, Blacksburg, VA, 24060, USA

**Keywords:** *Babesia* spp., Molecular surveillance, *Theileria* spp., *Theileria orientalis*, White-tailed deer, Tick-borne pathogens

## Abstract

In 2017, clinical disease and mortality in cattle associated with *Theileria orientalis* Ikeda was reported in Virginia, U.S. The exotic tick, *Haemaphysalis longicornis,* is a competent vector for this species. White-tailed deer (*Odocoileus virginianus*) are commonly infested with *H. longicornis* in the eastern U.S. and are also infected with several genotypes of piroplasms such as a *Theileria* sp. (often called *Theileria cervi-*like), *Babesia odocoilei*, and *Babesia* sp. H10. However, it is currently unknown if deer are susceptible to *T. orientalis* and can act as potential hosts. In this study, we tested 552 white-tailed deer samples from the southeastern U.S. to determine the presence of *T. orientalis*. We used a PCR-RFLP to test 293 (53%) of these samples to distinguish between piroplasm genera. A total of 189 white-tailed deer were positive with *Theileria,* 47 were positive with *Babesia*, and 57 did not amplify. Because this assay does not determine species, we sequenced 30 random samples targeting a fragment of the 18S rRNA gene. Although a high diversity of *Theileria* and *Babesia* spp. were detected, none were *T. orientalis*. All 552 samples were then screened with a *T. orientalis* specific real-time PCR protocol, but none were positive for *T. orientalis.* Our data suggests that white-tailed deer are commonly infected with piroplasm species but not *T. orientalis*.

## Highlights

• Piroplasm parasites of 552 white-tailed deer were surveyed in the Eastern U.S.
• A high prevalence and diversity of *Theileria* and *Babesia* species were observed.
• No infections with exotic *Theileria orientalis* Ikeda was detected in white-tailed deer.

## Graphical abstract

**Figure:**
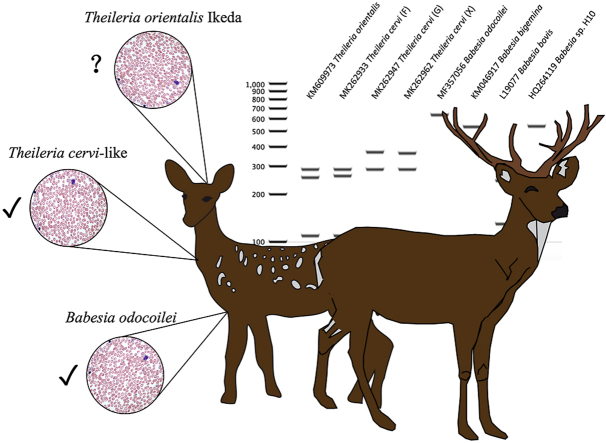

## 1 Introduction

Wildlife can maintain and transmit pathogens relevant to human and animal health. These shared pathogens may be of concern to the conservation of threatened or endangered species and can threaten food security (Wiethoelter et al., 2015). Recently, *Theileria orientalis* Ikeda genotype, an exotic vector-borne intraerythrocytic parasite, has been reported in cattle herds in Virginia, U.S. where it has been responsible for the morbidity and mortality of cattle (Oakes et al., 2019, Oakes et al., 2022). Additionally, detections of *T. orientalis* Ikeda have also been made in cattle in West Virginia, Tennessee, Pennsylvania, North Carolina, Kentucky, and Kansas (Virginia Tech Animal Laboratory Services, 2022). As the causative agent of bovine theileriosis, *T. orientalis* Ikeda has been responsible for significant economic losses in Asia, New Zealand, and Australia and presents a major concern to the cattle industry in the U.S. (Watts et al., 2016). The exotic Asian longhorned tick, *Haemaphysalis longicornis,* is a confirmed vector for this parasite and *T. orientalis* Ikeda has been detected in both cattle and host-seeking *H. longicornis* (Thompson et al., 2020b; Dinkel et al., 2021). Archived tick specimens reveal that the earliest detection of the Asian longhorned tick in the U.S. was in 2010 on a white-tailed deer from West Virginia (Thompson et al., 2022). Since then, the Asian longhorned tick has since been detected in multiple eastern states including those reporting *T. orientalis* Ikeda infections in cattle. Due to the detection in 2010, it is unknown if the introduction of *T. orientalis* Ikeda to the U.S. is a recent development.

*Theileria orientalis* Ikeda is one of many genotypes in the *T. orientalis* species complex (Watts et al., 2016). In addition to the Ikeda genotype, the Buffeli and Chitose genotypes have also been detected in cattle within the U.S. While there is currently little information regarding *T. orientalis* Chitose in cattle from Virginia; *T. orientalis* Buffeli, which is considered to be less pathogenic than the Ikeda genotype, was associated with mortality of cattle in Missouri (Stockham et al., 2000; Oakes et al., 2022). Other poorly characterized *Theileria* spp. have been reported in cattle from Texas in 1979 and 1993, as well as from North Carolina in 1995. Later analysis of the 18S rRNA gene revealed that some of these reports are most similar to *T. orientalis* Buffeli indicating a potentially widespread geographic and temporal distribution of this group of parasites (Chae et al., 1998).

Within the U.S., white-tailed deer (*Odocoileus virginianus*) are important hosts for the exotic Asian longhorned tick and may act as local and regional disseminators of the tick (Tufts et al., 2019; Thompson et al., 2022; White et al., 2021). White-tailed deer are also hosts for a diversity of piroplasm parasites closely related to *T. orientalis*, such as *Theileria cervi*-like and *Babesia odocoilei* (Davidson et al., 1983; Waldrup et al., 1992; Cauvin et al., 2019)*.* Some *Theileria* strains can cause clinical disease in deer (Yabsley et al., 2005; Wood et al., 2013). However, it is currently unknown if white-tailed deer are susceptible to *T. orientalis* genotypes. Therefore, we conducted surveillance using molecular techniques to investigate the presence of *T. orientalis* in white-tailed deer in the eastern U.S. We included a large number of white-tailed deer from Virginia, U.S. due to this area being a focal point for cattle infected with *T. orientalis* Ikeda (Virginia Tech Animal Laboratory Services, 2022)*.* Given the overlap of pathogens and vectors between deer and cattle, it is important to investigate the potential of white-tailed deer (and other wild ruminants) to become infected with *T. orientalis* and potentially contribute to maintaining both a pathogen and its vector that are important to livestock health.

## 2 Materials and methods

We opportunistically tested archived white-tailed deer blood samples collected during herd health checks and white-tailed deer mortality investigations submitted to the Southeastern Cooperative Wildlife Disease Study (SCWDS) at the University of Georgia. Given the historic and geographic spread of bovine theileriosis cases in the U.S., samples prior to the 2017 *T. orientalis* Ikeda mortality event as well as samples from other states were included in the study. Additional samples were collected from hunter-harvested deer in Virginia in 2018 and 2019 (Table 1). Asian longhorned tick infestation status for all blood samples received during this study is unknown. Genomic DNA from the samples was extracted using the Qiagen DNeasy® Blood and Tissue Kit (Hilden, Germany) following the manufacturer protocols and a ∼420 bp fragment of the 18S rRNA gene was amplified using PCR as described by da Silveira et al. (2011). Because of the high prevalence of piroplasms detected using PCR, sequencing was not a viable route to conduct this surveillance study. Using protocols in Thompson et al. (2020a), we developed a restriction fragment length polymorphism (RFLP) assay of this 18S rRNA amplicon using the *RsaI* (5′GT/AC3′) restriction enzyme (ThermoFisher Scientific, Waltham, MA). In short, we queried sequences of *Theileria* spp. and *Babesia* spp. found in the U.S. from GenBank. In Geneious Prime (Auckland, New Zealand, https://www.geneious.com), all sequences were aligned, filtered, and trimmed to contain the same 18S rRNA region that would amplify if using the primers described by da Silveira et al. (2011). Sequences were then experimentally digested in Geneious Prime with commercially available restriction enzymes to determine the best candidate for laboratory analysis. This PCR-RFPL assay distinguishes between the *Babesia* and *Theileria* genera, thus is a more rapid and cost-effective approach to screening when compared to sequencing. To confirm efficacy of this protocol, 30 randomly selected samples were selected (20 with a *Theileria* sp. RFLP and 10 with a *Babesia* sp. RFLP) and sequenced to confirm PCR-RFLP results. All samples were then screened using a real-time PCR assay (Dinkel et al., 2021) which is specific to the major piroplasm surface protein (*MPSP*) of the *Theileria orientalis* species complex (can detect Buffeli, Chitose, and Ikeda subtypes).

**Table 1:** Summary data of piroplasms in white-tailed deer (*Odocoileus virginianus*) sampled by state, year, and testing method.

State	No. of Deer Sampled	Years of Sample Collection	Piroplasm PC^a^-RFLP^b^				*Theileria orientalis* RT-PCR	
			No. Tested (%)^a^	PCR Negative	*Theileria* sp. positive	*Babesia* sp. positive	No. Tested	No. Positive
**Alabama**	10	2006	10 (100%)	0	9 (90%)	1 (10%)	10 (100%)	0
**Arkansas**	7	2001	7 (100%)	0	7 (100%)	0	7 (100%)	0
**Georgia**	21	2006–2018	16 (76%)	3 (19%)	12 (75%)	1 (6%)	21 (100%)	0
**Kentucky**	10	2018	10 (100%)	10 (100%)	0	0	10 (100%)	0
**Maryland**	7	2002	7 (100%)	1 (14%)	0	6 (86%)	7 (100%)	0
**Mississippi**	5	2001	5 (100%)	0	3 (60%)	2 (40%)	5 (100%)	0
**Nebraska**	1	2018	1 (100%)	1 (100%)	0	0	1 (100%)	0
**North Carolina**	1	2018	1 (100%)	0	1 (100%)	0	1 (100%)	0
**Oklahoma**	53	2016–2018	53 (100%)	3 (6%)	49 (92%)	1 (2%)	53 (100%)	0
**Texas**	1	2018	1 (100%)	0	1 (100%)	0	1 (100%)	0
**Virginia**	416	2018–2019	162 (39%)	39 (24%)	87 (53%)	36 (23%)	416 (100%)	0
**West Virginia**	20	2011^a^- 2018	20 (100%)	0	20 (100%)	0	20 (100%)	0
**Total**	552		293 (53%)	57 (20%)	189 (65%)	47 (16^b^)	552 (100%)	0

a Not all deer were initially screened with the Piroplasm PCR-RFLP; however, all deer were screened with the *Theileria orientialis*-specific PCR.

b RFLP, Restriction Fragment Length Polymorphisms.

## 3 Results

A total of 552 white-tailed deer blood samples were screened (Table 1). Of these, 427 (77%) were collected from deer from Virginia and West Virginia in regions of the respective state with known *T. orientalis* Ikeda infections and *H. longicornis* infestations (Leonhardt, 2020; Thompson et al., 2020b; Oakes et al., 2022). The other 125 samples were opportunistically included from other states (Table 1). Of the 552 samples, 293 (53%) were screened with the PCR-RFLP method as a pilot study to determine the prevalence of the two piroplasm genera. Of these, 234 (79.9%) were PCR-positive and, 189 (81%) were determined to have *Theileria* infections while 47 (20%) were positive for a *Babesia* using the RFLP assay (Table 1). Sequence analysis of 30 (13%) random samples from this pilot study revealed that the PCR-RFLP *Theileria*-positive deer were infected with *Theileria* spp. related to the *T. cervi-*like species complex (96.3–100% identical to JX274294; 99.3–99.5% identical MW008534; and 98.9–99.1% identical to KF790922). The PCR-RFLP *Babesia-*positive deer were infected with different *Babesia* spp. previously reported in white-tailed deer (99.1–100% identical to *B. odocoilei* (MW480558) and 98–99.8% identical to *Babesia* sp. BCS-2013b (KC162915)). All unique sequences were deposited into Genbank under accession numbers OL435131 – OL435142. Due to the inability to determine co-infections of parasites within deer samples and parasite species via the PCR-RFLP without sequencing as well as the high prevalence and diversity of *Theileria* spp., we screened all 552 samples for *T. orientalis* using the real-time PCR assay. None of the white-tailed deer from this study were positive for any genotype of *T. orientalis*.

## 4 Discussion

Overall, our study showed that there was a high prevalence and diversity of piroplasms in white-tailed deer; however, none of the tested samples were positive for any genotype of *T. orientalis* when sequenced or screened using the real-time PCR assay. Given the high number of deer sampled from an area with known *T. orientalis* Ikeda infections, these data suggest that white-tailed deer are not suitable hosts for *T. orientalis*. These results are consistent with previous surveillance studies that failed to detect *T. orientalis* in cervids within the other established ranges of *H. longicornis* and *T. orientalis* Ikeda (Li et al., 2014; Huaman et al., 2021; Mohamed Moustafa et al., 2021). While our study focused on white-tailed deer, there are other free-ranging ungulates, specifically bovids such as goats, bison (*Bison bison*), or nilgai (*Boselaphus tragocamelus*) that could be susceptible to *T. orientalis*. Should the Asian longhorned tick continue to spread to the western U.S. where these other potential species are, surveillance for this parasite would be warranted*.* Experimental studies investigating the ability of other wild ungulates to be infected with *T. orientalis* should be pursued to better understand the risk this parasite may pose to wildlife and livestock health.

The other piroplasm parasites detected from the deer in this study are of significant health interest. *Babesia odocoilei* is a pathogen of elk (*Cervus canadensis*) and reindeer (*Rangifer tarandus*) and was recently recognized as an emerging zoonotic pathogen (Scott et al., 2021). Furthermore, *B. odocoilei* and members of the *T. cervi*-like species complex have also been responsible for mortalities in juvenile and farmed white-tailed deer and mule deer (*Odocoileus hemionus*) throughout the eastern U.S. (Yabsley et al., 2005; Wood et al., 2013; Mathieu et al., 2018). Unfortunately, there are few data about the diversity and health implications of native cervid piroplasm parasites in the U.S. warranting a need for more studies on the characterization and pathogenesis of these parasites as well as comprehensive review on the topic.

## 5 Conclusion

The present study investigated the piroplasm parasite prevalence and diversity in white-tailed deer from the eastern U.S. The primary goal of the study was to determine if white-tailed deer are infected with exotic *Therileria orientalis* Ikeda, a parasite of significant agricultural concern. None of the deer screened in this study were determined to be infected with *T. orientalis*, including many deer tested in areas where the pathogen or vector are known to occur, suggesting that they may not be suitable hosts for this parasite. However, additional research is needed to better understand the possible maintenance of this exotic parasite in wild ungulates and risks to cattle populations in the U.S.

## Declaration of competing interest

The authors declare that they have no known competing financial interests or personal relationships that could have appeared to influence the work reported in this paper.
